# User experiences with food storage in a meatsafe (mesh-covered food storage cabinet) in an informal settlement in Dhaka, Bangladesh

**DOI:** 10.1186/s41182-026-00983-x

**Published:** 2026-06-05

**Authors:** Musarrat Jabeen Rahman, Syeda Nurunnahar, Mahbubur Rahman, Laura H. Kwong, Peter J. Winch

**Affiliations:** 1 Social and Behavioral Interventions Program, Department of International Health, Johns Hopkins Bloomberg School of Public Health, Baltimore, MD 21205 USA; 2 Environmental Health and WASH, Health Systems and Population Studies Division, International Centre for Diarrhoeal Disease Research, Bangladesh (Icddr,B), Dhaka, 1212 Bangladesh; 3 Global Health and Migration Unit, Department of Women’s and Children’s Health, Uppsala University, Uppsala, Sweden; 4 Environmental Health Sciences, Global Environmental Health, University of California, Berkeley, CA 94720 USA

**Keywords:** Food storage, Informal settlements, Bangladesh, Food hygiene, Diarrhoeal disease, *Escherichia coli*, Qualitative research, Behavior change communication, Urban health

## Abstract

**Background:**

Food hygiene is a critical but underemphasized link in fecal–oral disease transmission in dense informal settlements. A mesh-walled meatsafe, designed to exclude insects, animals, and young children while allowing ventilation, was distributed in a randomized controlled trial in Korail, Dhaka. Despite near-universal use (87.7–99.2% of intervention households storing cooked food across five spot-check rounds), the trial detected no reductions in food contamination or child diarrhoea. This qualitative substudy investigated what technological, psychosocial, and contextual factors limited the meatsafe’s protective potential despite high observed use.

**Methods:**

Guided by the Integrated Behavioral Model for Water, Sanitation, and Hygiene (IBM-WASH), we conducted 16 in-depth interviews with primary caregivers, purposively sampled to capture variation in meatsafe condition, maintenance, and food contamination profiles, and triangulated findings against spot-check records. Transcripts were thematically coded across IBM-WASH domains.

**Results:**

Barriers preventing correct device use (fidelity barriers) included a steam-spoilage belief leading caregivers to cool food uncovered before meatsafe placement, rexine liner warping reinforcing cooling delays, and cumulative hardware friction from frame instability, latch weakness, and size constraints. Barriers that operated outside the scope of the intervention (beyond-fidelity barriers) included energy scarcity enforcing batch cooking with extended storage, monsoon flooding contaminating cooking spaces, seasonal displacement disrupting routines, and contested childcare authority limiting maternal protective behavior. The uncovered cooling period was invisible to spot-check monitoring yet represented the highest-risk contamination window.

**Conclusions:**

Near-universal meatsafe use without contamination reductions is explained by a behavioral gap invisible to spot-check monitoring: households stored food only after an uncovered cooling period. The meatsafe addressed only one F-diagram pathway; other fecal–oral routes remained uncontrolled. Sustainable reduction of foodborne enteric disease requires co-designed hardware addressing the full causal pathway, behavior change communication directly confronting the steam-spoilage belief, and concurrent structural investment in energy access, drainage, and pest control.

**Supplementary Information:**

The online version contains supplementary material available at https://doi.org/10.1186/s41182-026-00983-x.

## Introduction

Food hygiene occupies a pivotal node in the fecal–oral transmission schematic, the F-diagram [37], yet it received relatively little emphasis in recent WASH interventions [13, 18, 23]. A growing body of evidence nevertheless underscores its public health relevance. In urban Bangladesh, children whose households left stored food uncovered had 6.16-fold higher odds of diarrhoea than those whose food was covered [36]. In both rural and urban Bangladeshi settings, consumption of food with high fecal bacterial counts elevated the risk of diarrhoea within 24 h [15], and the presence of *Escherichia coli* (*E. coli*) in food doubled the odds of bloody diarrhoea [29].

Trials targeting complementary food hygiene have incorporated varying levels of “hardware” and “software” (behavior change communication, BCC). *Banja la Ukhondo* in Malawi delivered 33 participatory BCC sessions over 31 weeks, guiding households to fabricate tippy-taps, dish racks, and pot lids; despite providing no hardware, it reduced self-reported diarrhoea by 13 percentage points [3, 25]. A BCC-intensive hygiene intervention based on hazard analysis and critical control point (HACCP) principles in Bamako, Mali promoted handwashing, utensil cleaning, and reheating during daily home visits over 4 weeks, and achieved significant reductions in fecal *E. coli* forms at critical control points [33]. A similar BCC-only trial in low-income neighborhoods of Dhaka, Bangladesh provided 4 weeks of daily household visits promoting the same food hygiene practices, which virtually eliminated *E. coli*, *Streptococci*, and *Clostridium perfringens* in weaning foods, with effects sustained for 3 months after the intervention [16]. While interventions that rely primarily on intensive BCC through frequent promoter contact might reduce contamination levels and diarrhoeal incidences in the short run, these labor-intensive approaches are not scalable or sustainable [24].

Minimal BCC, coupled with hardware support, may have potential for sustained habit formation [8, 19]. Rather than instructing caregivers step by step with complex behavioral messages over multiple visits, the idea of minimal BCC draws on behavioral economics principles of “nudges,” where subtle environmental cues help shape everyday choices. Nudges have proven effective in shifting related practices in other domains: for instance, painted footprints leading to school handwashing stations increased handwashing [7, 10], and children served meals on plates printed with dietary guidance made healthier food selections [2].

Formative research in rural Bangladesh revealed that caregivers typically cook once per day, store food for 4–8 h, and dislike tight lids that trap steam when food is cooling. Many expressed enthusiasm for a meatsafe, a metal mesh-walled cabinet that excludes insects, domestic animals, and probing of stored foods by young children while allowing food to cool; users reported less spoilage and neighbors inquired where they could obtain one [30].

The meatsafe evaluated in this trial is a lightweight metal cabinet with mesh-screen walls on three sides, a solid metal back panel, a solid metal top and base, two hinged doors secured by a small latch, and three interior shelves. The mesh allows air circulation, enabling steam to escape from freshly cooked food, while the screen is intended to exclude flies, cockroaches, and rodents. The theoretical protective mechanism is physical exclusion of insect and animal vectors at the point of food storage, reducing post-cooking fecal–oral contamination without requiring behavior changes beyond placing cooked food inside and closing the door. No prior controlled efficacy data existed for this specific commercial design at the time the trial was initiated.

Accordingly, we evaluated the meatsafe in a randomized controlled trial in Korail, Dhaka’s largest informal settlement.

Five spot-check rounds over 2 months documented near-universal meatsafe usage for cooked food: 87.7–99.2% of intervention households stored cooked food in the project meatsafe (reported in full in Rahman et al., in press [31]. Among cooked foods present in the project meatsafe, the proportion fully covered rose from 66.9% at PI-1 to 88.4% at PI-5.

Nevertheless, the endline assessment detected no reduction in *E. coli* among foods stored >4 h, nor in caregiver-reported child diarrhoea.

The Integrated Behavioral Model for Water, Sanitation, and Hygiene (IBM-WASH) provides the analytical framework for this study [8]. IBM-WASH organizes determinants of WASH-related behaviors across three domains––technological (features of the hardware or infrastructure), psychosocial (beliefs, norms, attitudes, and self-efficacy), and contextual (physical, social, and structural environment)––and five levels of influence: societal/structural, community, household/interpersonal, individual, and habitual. The framework was designed specifically for infrastructure-restricted settings and is well suited to examining why a hardware intervention may fail to generate the behavioral changes necessary for health impact.

We designed this qualitative substudy prospectively, alongside the parent trial, as a planned mechanism-of-impact inquiry to explore how households in Korail integrated the meatsafe into daily food storage, cooking, and caregiving routines, and what technological, psychosocial, and contextual factors shaped its use. Once the endline assessment documented near-universal uptake without corresponding reductions in food contamination or child diarrhoea, the analytic focus sharpened into one question: what barriers constrained the meatsafe’s protective potential despite high observed use?

## Methods

### Intervention description

This manuscript reports the qualitative substudy nested within the parent RCT. Details of the parent trial’s randomization, allocation concealment, blinding, and registration are reported in the companion quantitative manuscript [31]. The qualitative inquiry did not involve randomization and required no independent trial registration.

The intervention supplied a low-cost meatsafe plus one 15–20-min session of BCC at the first post-intervention visit, when the meatsafes were provided to the household. Our theory of change was that giving caregivers a cabinet intended to ventilate steam and keep out flies and simple instructions for use and basic food hygiene should prompt immediate, routine storage of cooked foods, reducing fecal contamination and lowering childhood diarrhoea. Two posters accompanied it: one on correct meatsafe use (immediate storage, regular cleaning, and keeping the cabinet dry) and one on general food hygiene behaviors (thorough cooking, handwashing, and boiling water). The general food hygiene recommendations were drawn from an intervention in Nepal [9]. The current intervention adapted visual design and terminology to the local context in consultation with icddr,b field staff. The core behavioral recommendations (thorough cooking, immediate covered storage, regular meatsafe cleaning, handwashing, and boiling water for drinking) were adopted without substantive modification.

The BCC package promoted immediate covered storage but did not directly address whether food should be enclosed while still hot. At the time of intervention design, the available evidence base on storage temperature and microbial safety did not establish that immediate sealing of hot food in a meatsafe was microbiologically safer than allowing brief uncovered cooling before storage. In the absence of supporting evidence, the design team did not include messaging that contradicted the locally held belief that enclosing hot food traps steam and accelerates spoilage. The implications of this design choice for the trial’s null result are considered in Discussion.

Field staff visited households fortnightly to collect RCT food samples and inspect meatsafe condition.

### Study setting and participants

We collected data from 15 November 2024 to 15 December 2024 in Korail, Dhaka’s largest informal settlement [27, 32]. The neighborhood is characterized by its mix of corrugated iron shed structures and a few buildings with brick walls [4]. Access to basic water and sanitation services is limited [1, 35], while inadequate drainage and the consequent spread of contaminated floodwater and flooding contribute additional contamination risk [27]. Access to natural gas for cooking arrives late at night, so some households purchase firewood or propane for daytime cooking [5].

All participants were recruited from the parent RCT’s intervention arm. For this study, “primary caregiver” was defined as the adult household member bearing primary responsibility for preparing and storing food for the index child. In all 16 interviewed households, the primary caregiver was an adult woman (mother, grandmother, or mother-in-law of the index child).

### Sampling strategy

We employed purposive sampling to capture a range of meatsafe conditions, maintenance profiles, and food contamination levels. Using fortnightly spot-check data and laboratory results from the parent RCT, we generated a candidate list of intervention households meeting one or more criteria across PI-1–PI-5: (a) cleanliness rated “some dirt” or “considerable dirt”; (b) door functionality problems; (c) screen holes; or (d) any *E. coli* detected in stored food (including low-level 10 or more CFU/g and high-level 100 or more CFU/g). Of 145 intervention households, 133 (92%) met at least one criterion. Within this pool, we prioritized households with high-level *E. coli* contamination or documented structural or cleanliness problems.

Research assistants approached households from this prioritized list in person and by telephone. Thirteen households from the issue-identified pool consented to participate. To broaden the range of experiences represented, we also recruited 3 households with no recorded maintenance issues and no *E. coli* detection, for a total of 16 in-depth interviews. No household contributed more than one interview.

The resulting sample captured deliberate variation across maintenance, contamination, and use profiles (Table 1). All 16 households had cooked food observed inside the meatsafe at every post-intervention visit; three had one or two missed visits due to temporary absence. Among interviewed households, 10 had cleanliness problems at one or more visits, 2 had door functionality problems, and 1 had screen holes. Six households had high-level *E. coli* contamination (100 or more CFU/g), 3 had low-level contamination only, and 7 had no *E. coli* detected. The three no-issue households enabled comparison between households with and without documented problems.

**Table 1:** Characteristics, meatsafe condition, and food contamination profiles of qualitative substudy participants (*N* = 16)

Participant	Characteristics				Meatsafe condition (any PI visit)^a^				*E. coli* contamination^b^
ID	Age (yrs)	Relation to index child	HH size	Education	PI visits attended	Door problem	Screen holes	Dirt observed	Category
P01	25	Mother	3	6 yrs	5	✓	✓	–	None
P02	30	Mother	5	11 yrs	5	✓	–	✓	High
P03	20	Mother	4	Graduate	5	–	–	✓	None
P04	20	Mother	3	Graduate	5	–	–	✓	High
P05	40	Mother	5	No education	5	–	–	✓	High
P06	20	Mother	3	Higher secondary	5	–	–	–	High
P07	26	Aunt	3	6 yrs	5	–	–	✓	None
P08	18	Mother	5	8 yrs	4^c^	–	–	✓	High
P09	18	Mother	5	Graduate	5	–	–	–	None
P10	22	Mother	3	6 yrs	5	–	–	✓	High
P11	25	Mother	4	6 yrs	3^c^	–	–	✓	Low
P12	33	Mother	5	No education	5	–	–	✓	None
P13	22	Mother	3	11 yrs	5	–	–	–	None
P14	28	Mother	3	Graduate	4^c^	–	–	✓	Low
P15	20	Mother	4	6 yrs	5	–	–	–	Low
P16	20	Mother	3	Secondary	5	–	–	–	None

Participant identifier assigned in ascending household ID order. Age, relationship to index child, household size, and educational attainment are drawn from parent randomized controlled trial baseline records for the 16 interviewed households

*HH* household, *PI* post-intervention, *yrs* years of completed schooling, *graduate* graduate and above

^a^✓ = Observed at one or more of five post-intervention spot-check visits; – = not observed at any visit. All 16 households had cooked food observed inside the meatsafe at every attended visit

^b^*E. coli* contamination category based on food samples collected at baseline and all post-intervention visits. None = no *E. coli* detected (<10 CFU/g) at any visit; Low = ≥10 CFU/g detected but never ≥100 CFU/g; High = ≥100 CFU/g (high-level) detected at one or more visits. CFU/g = colony-forming units per gram

^c^Household had one (P08, P14) or two (P11) missed post-intervention visits due to temporary absence from the settlement

Interviews continued until thematic saturation was reached. New themes continued to emerge through interview 12; interviews 13–16 yielded only minor elaborations on established themes, indicating that thematic saturation had been reached.

### Data collection and analysis

Two Bengali-speaking research assistants (RAs) conducted in-home, semi-structured interviews lasting 30–60 min. MJR supervised data collection, held regular debriefing sessions with RAs, reviewed field notes and memos, and checked all transcripts for completeness, but was not present during main interviews. Both RAs held bachelor’s degrees in public health or social science and had prior qualitative research experience in community health settings. Before data collection, RAs completed a 3-day training covering the interview guide, probing techniques, audio recording, field note conventions, and ethical protocols including informed consent and confidentiality. All interviews took place in the participant’s primary domestic space, typically a single- or two-room corrugated iron structure containing the meatsafe.

RAs recorded observational notes on meatsafe condition, placement, shelf contents, and visible adaptations (liners, stabilizing bricks, taped seams) in structured field notes immediately after each interview. These notes served as a triangulation source alongside transcripts and spot-check data.

All interviews were audio-recorded with participants’ informed consent. The interview guide was developed iteratively by MJR drawing on IBM-WASH domain definitions and formative knowledge of Korail, then reviewed by all co-authors. It was pilot tested in two households in an adjacent non-intervention area of Korail with similar socioeconomic and housing characteristics, not enrolled in the parent RCT. Pilot interviews were conducted by MJR together with one RA as part of RA training and guide refinement. Three probes identified as producing confusion or minimal elaboration were reworded before main data collection began. Pilot data are not included in the findings. The final guide is shown in Supplementary File 1. Recordings were transcribed verbatim, translated into English, and reviewed by bilingual researchers for fidelity.

Two coders (MJR and SN) independently read each transcript and applied a first-pass code of “barrier” to any segment in which a participant expressed a negative experience, struggle, concern, or opinion related to food storage, meatsafe use, or adherence to recommended practices, using NVivo version 14 (Lumivero, Denver, CO, USA). Coders consulted each other to resolve any ambiguities in code application as they arose. Coded segments were sorted into the three IBM-WASH domains (technological, psychosocial, contextual) and grouped into subthemes through iterative comparison. A quote-to-code-to-theme mapping is provided in Supplementary Table 1. The five IBM-WASH levels of influence (societal/structural, community, household/interpersonal, individual, habitual) were not applied as a systematic analytical axis; 16 interviews do not generate sufficient coverage to populate level-specific cells across all three domains with evidentiary confidence, so the domains served as the organizing structure and level attributions appear only where the data directly support them.

Spot-check data were analyzed descriptively using frequencies and column percentages (Stata version 18, StataCorp, College Station, TX, USA), summarized in Table 2, and used as both the sampling frame and a triangulation source against interview-derived themes. No inferential analyses were conducted within this substudy; quantitative trial outcomes are reported in the companion RCT manuscript [31]. This study was reported in accordance with the Consolidated Criteria for Reporting Qualitative Research (COREQ); a completed 32-item checklist is provided in Supplementary File 2.

**Table 2:** Meatsafe condition observed across five post-intervention visits in the parent trial

Category	Condition	PI-1 (*N* = 141)	PI-2 (*N* = 137)	PI-3 (*N* = 137)	PI-4 (*N* = 136)	PI-5 (*N* = 133)	Total (*N* = 684)
Door condition	Closes well	124 (87.9%)	132 (96.4%)	134 (97.8%)	135 (99.3%)	132 (99.3%)	657 (96.1%)
	Some difficulty closing	6 (4.3%)	3 (2.2%)	2 (1.5%)	0 (0.0%)	0 (0.0%)	11 (1.6%)
	Does not close	11 (7.8%)	2 (1.5%)	1 (0.7%)	1 (0.7%)	1 (0.8%)	16 (2.3%)
Cleanliness	Very clean	112 (79.4%)	92 (67.2%)	107 (78.1%)	110 (80.9%)	111 (83.5%)	532 (77.8%)
	Some dirt	27 (19.1%)	43 (31.4%)	28 (20.4%)	25 (18.4%)	21 (15.8%)	144 (21.1%)
	Considerable dirt	2 (1.4%)	2 (1.5%)	2 (1.5%)	1 (0.7%)	1 (0.8%)	8 (1.2%)
Screen condition	No holes	139 (98.6%)	137 (100.0%)	137 (100.0%)	136 (100.0%)	131 (98.5%)	680 (99.4%)
	One hole	1 (0.7%)	0 (0.0%)	0 (0.0%)	0 (0.0%)	2 (1.5%)	3 (0.4%)
	Multiple holes	1 (0.7%)	0 (0.0%)	0 (0.0%)	0 (0.0%)	0 (0.0%)	1 (0.1%)

Values are *N* (column %). PI = post-intervention visit. *N* represents the total number of households observed at each visit. Visit-level sample sizes range from 133 to 141 (out of 145 enrolled households) due to missing observations, including cases where respondents were not present at the time of data collection. The total column reflects pooled observations across all five visits (*N* = 684). Data are drawn from the full intervention arm of the parent randomized controlled trial and are not limited to the 16 households that participated in qualitative interviews; they are presented as the quantitative backdrop against which qualitative findings are interpreted. PI-1–PI-5 = post-intervention visits 1–5

Cleanliness ratings used a structured visual rubric: “very clean” = no visible dirt or insect debris; “some dirt” = visible residue on one or more surfaces; “considerable dirt” = accumulation on multiple surfaces or mold

### Reflexivity

MJR, who supervised data collection and led analysis, is a Bangladeshi public health researcher with prior icddr,b experience in Dhaka informal settlements. This brought not only advantages such as language, cultural familiarity, and rapport with field staff but also risks of over-familiarity. To manage these, MJR kept memos throughout coding, and the team (SN, PJW, LHK) met regularly to discuss and test emerging interpretations. Interview themes were compared against the 684 spot-check observations and RA field notes.

### Ethical considerations

This study was approved by the University of California, Berkeley Committee for Protection of Human Subjects (Protocol #2024–04-17365; approved 17 April 2024) and the Ethical and Research Review Committee of icddr,b (PR-24022; approved March 2024). Audio recordings were stored on password-protected devices, transcripts were de-identified before analysis, and no participant names appear in any study document. Participants were informed that participation was voluntary, that withdrawal carried no consequences, and that their responses would not affect their continued participation in the parent RCT. Written informed consent was obtained using Bengali-language forms signed by the participant and counter-signed by the interviewer as an independent witness, as specified in and approved by both IRB protocols as standard practice for community-based qualitative research at icddr,b. No minors were included.

## Results

The following results are organized by IBM-WASH domain—technological, psychosocial, and contextual—with quantitative spot-check data from all 145 intervention households integrated thematically where relevant as a population-level observational backdrop; full spot-check frequencies are summarized in Table 2. Sociodemographic characteristics, meatsafe condition profiles, and food contamination categories for the 16 qualitative participants are presented in Table 1.

### General food handling and storage practices

Prior to receiving the meatsafe, participants stored cooked food on the floor under plastic covers, in jute sacks, or on wooden racks, leaving food exposed to cats, cockroaches, and flies. Spot-check records from the parent trial confirmed that 87.7–99.2% of intervention households stored cooked food in the meatsafe across all five post-intervention visits, indicating near-universal device adoption [31] ; Table 2 captures cabinet condition only and does not include food storage prevalence data (see Table 1, footnote a, for the qualitative substudy sample specifically).

Furthermore, even though all participants received BCC guidance on correct meatsafe use, the in-depth interviews and field observations revealed that the device was adapted to broader household needs that extended beyond its intended function, and barriers across all three IBM-WASH domains constrained its protective potential despite high observed use.

### Technological dimension

#### Storage stability and structural support

The meatsafe’s lightweight design caused many users to report wobbling or shifting, particularly when full. Caregivers avoided heavier items (large pots of rice or dal) due to tipping risk, using the meatsafe only for lighter items and placing heavier pots on the floor. Most families stabilized the cabinet by placing bricks or boards underneath. One caregiver said:

We put bricks underneath, otherwise it shakes. The floor isn’t level, and when you open the door, it can tip forward. (P12)

Uneven or damp floors were noted in field observations of many of the households, consistent with corrugated iron shed construction and open drainage conditions in Korail, making stabilization a near-universal improvised challenge.

#### Latch weakness and structural gaps

Spot-check records documented high observed door functionality: 87.9% of doors closed well at PI-1, rising to 99.3% at PI-5, and 99.4% of screens had no holes across all visits (Table 2). Qualitative interviews, however, revealed that several households found the latch too small, poorly installed, or prone to breaking. Even when doors were nearly closed, a gap of two to three fingers remained sufficient for pest entry, and several households used cloth or tape to hold broken latches shut. Caregivers shared:

The latch needs to be fastened, but there are gaps. My husband put cello tape inside it to try to close them. All around the inside there are gaps. Even if I fasten the latch, there are still gaps. (P01)

My husband tried to tape some parts inside to make it tighter, but still, there’s a gap. Cockroaches sneak in at night. It would be better if it had a bigger, stronger latch, something that locked properly. (Composite from responses from P01, P02, P11)

Tape and cloth were used to reinforce the latch, but neither proved durable. Child tampering with the latch was also reported by several mothers, who expressed a need for child-resistant closures.

#### Size and multi-use storage needs

**Fig. 1:**
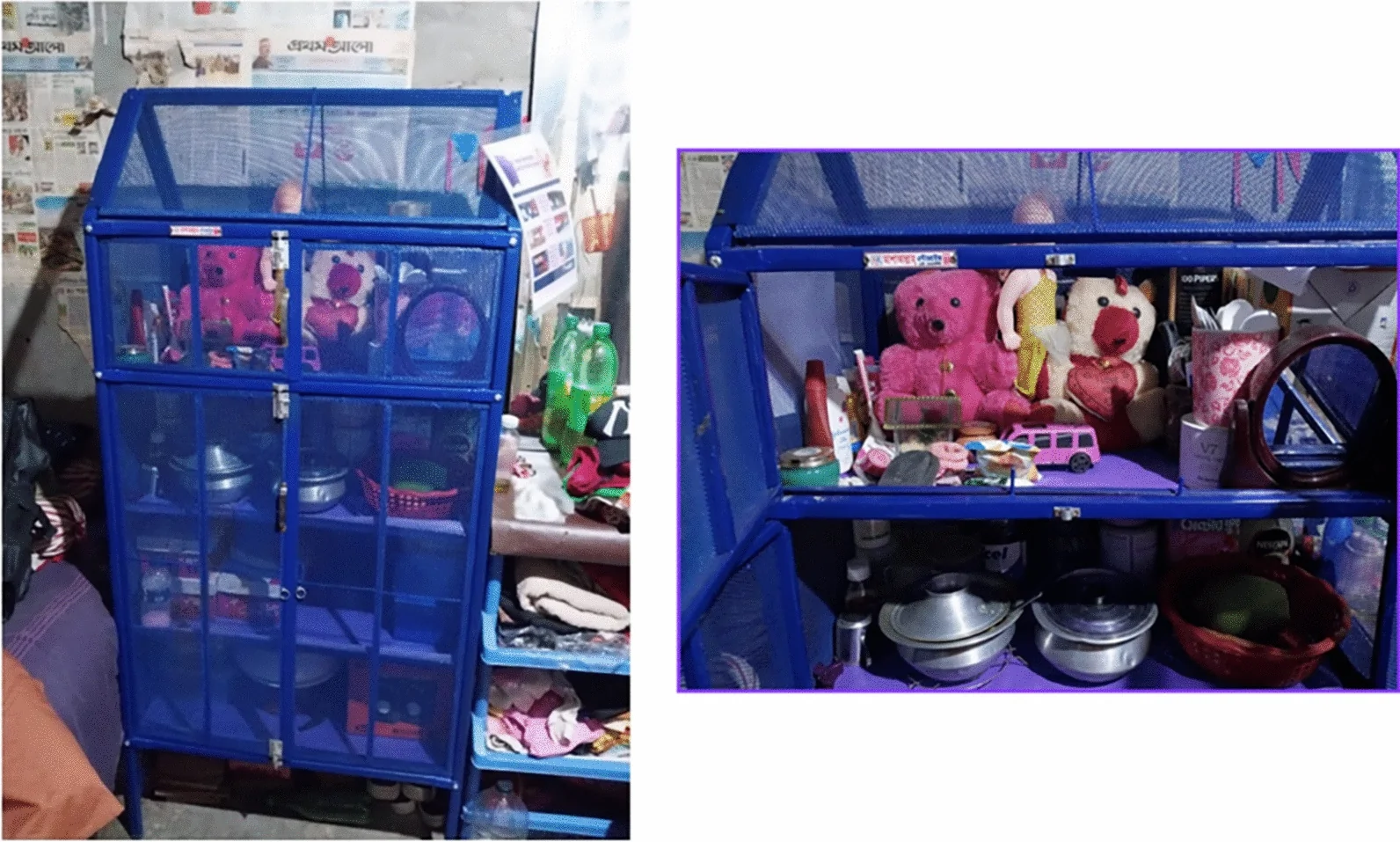
Meatsafe overloading: non-food and food items stored together. Photographs illustrate how households used the meatsafe to store multiple categories of items, including cooked food, uncooked food, utensils, powdered milk, spices, and personal items, resulting in overcrowding and reduced space for cooked food storage

Households routinely stored biscuits, raw vegetables, powdered milk, spices, oil bottles, and utensils alongside cooked food, and some repurposed the meatsafe for non-food items such as clothes, medicines, or baby supplies (Fig. 1). A mother of two explained:

We store many things in the same space, biscuits, onions, dry food, children’s chips. But we can’t fit everything. If it were bigger, we could keep large cooking pots inside, too. (Composite from responses from P01, P06)

#### Frequent cleaning but persistent dust

No microbial testing of the interior was conducted. Overall cleanliness was strong (77.8% “very clean” across all visits), dipping to 67.2% at PI-2 before recovering to 83.5% at PI-5 (Table 2). Despite active cleaning efforts, sharp edges and difficult-to-clean corners made sustained cleanliness challenging:

When we wipe it, our hands get cut from the edges. Dirt gets stuck in the corners. If we had a smoother, sturdier rack, it would help. Even after cleaning, dust collects again. (Composite from responses from P03, P14)

#### Missing liners and improvised solutions

**Fig. 2:**
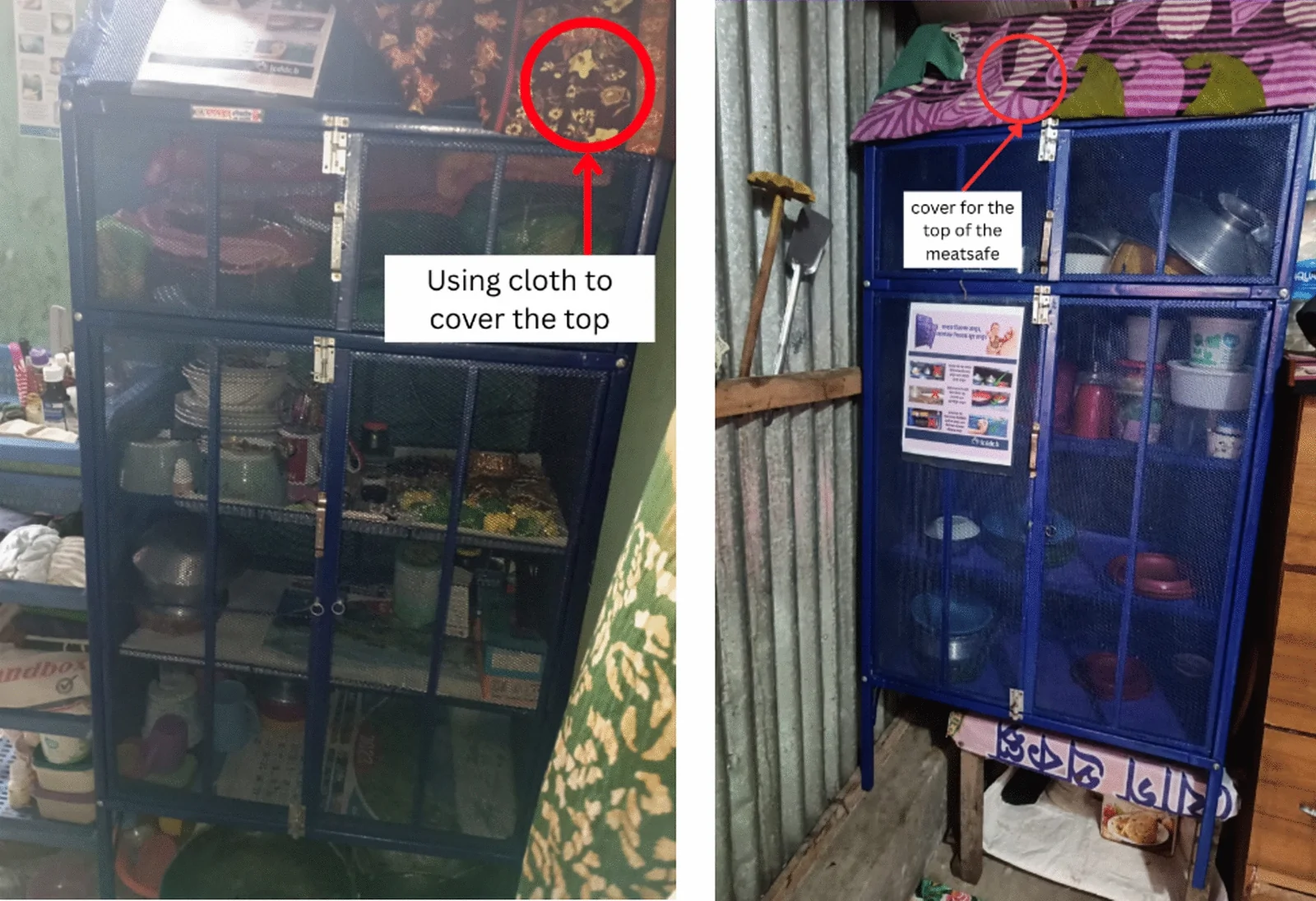
Meatsafe usage patterns: examples of using cloth to cover the top. Images show households placing cloth or rexine on top of the meatsafe to prevent dust accumulation and protect stored items, reflecting routine adaptations made to maintain cleanliness

Several households placed cloth on top of the meatsafe to prevent dust from settling on the surface and entering through the mesh (Fig. 2). Participants described a progression from newspaper (which tore when wet and attracted cockroaches) to cotton scarves and, ultimately, to rexine (an inexpensive PVC-coated fabric) as the preferred shelf liner. One caregiver said:

The newspaper liner tears when it gets wet. Then cockroaches come. So, I use cloth instead. I cut up my orna [traditional woman’s scarf] and put it on the shelves. (P04)

**Fig. 3:**
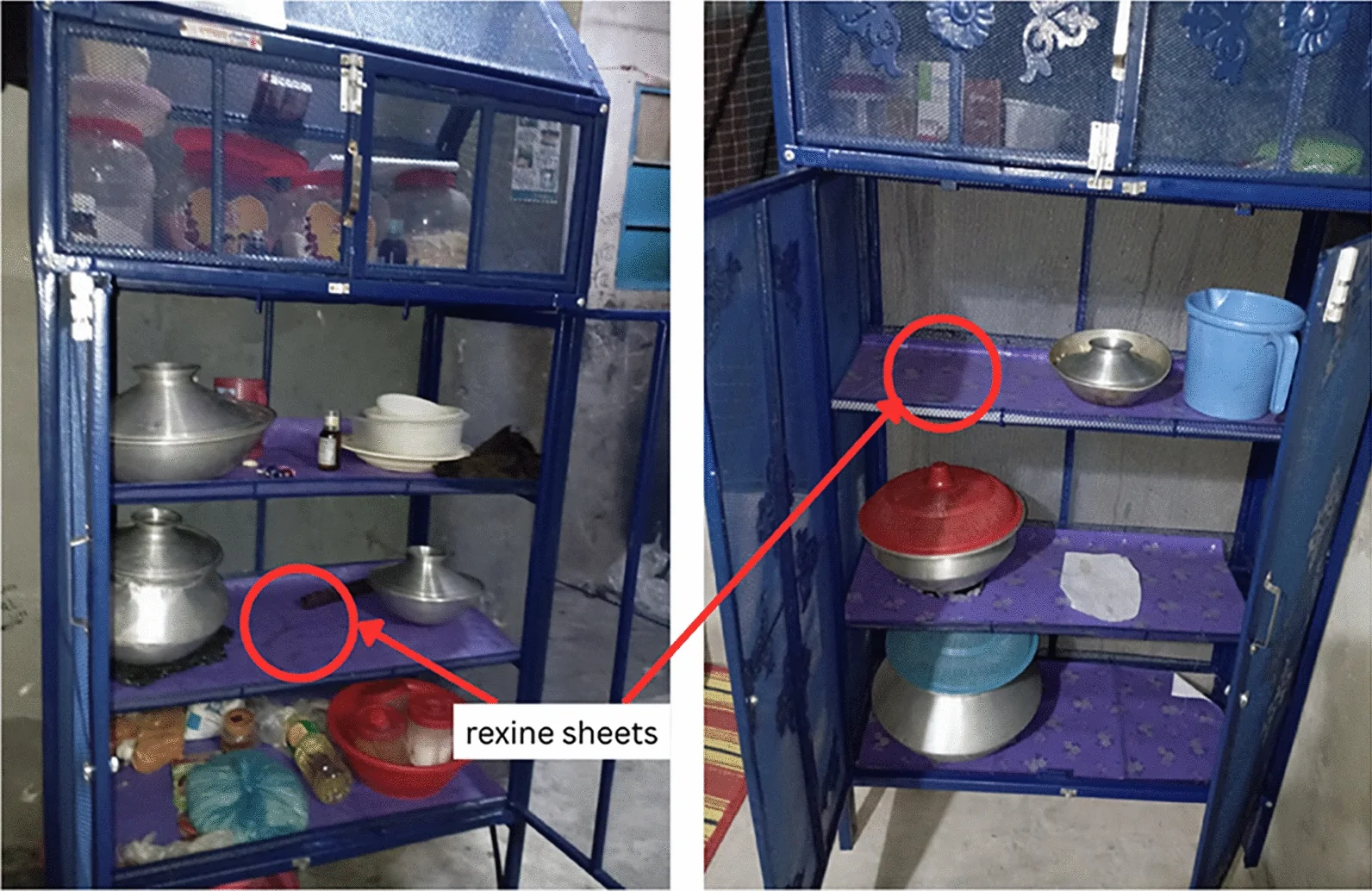
Rexine sheets to cover the base of the shelves. Images show rexine liners added by caregivers to protect the meatsafe shelves. Several households reported concerns about rexine warping or melting when placing hot pots inside the meatsafe

However, many participants feared that hot pots would warp or melt the rexine (Fig. 3), making them hesitant to store food immediately after cooking. This created a high-risk cooling window during which food sat uncovered:

If it were made of stronger material, I could store hot food without the rexine. Now, I have to let it cool for 7–8 minutes before putting it in. By then, flies might land on it. So, we place the food down on the floor and wait. When the pot cools down a bit, then we put it inside the meatsafe. (Composite from responses from P01, P02)

### Psychosocial dimension

#### Mental models of steam and food spoilage

Despite BCC messaging recommending immediate covered storage, participants consistently delayed storage, citing concerns that trapped steam accelerates spoilage:

If we touch the container and it is still warm, then storing it then will cause it to spoil. And so, we let the food cool down first. Then we store it, so it stays good. Because if you cover it immediately while it’s hot, moisture collects inside. That’s why I let it cool down a bit first. Then I cover it or store it. Otherwise, the rice turns yellow and sour. (Composite from responses from P06, P08, P16)

Condensation on the rexine liner, and then dust falling on that condensation, further reinforced this concern. At the individual IBM-WASH level, this concern created the uncovered cooling gap identified as the central mechanism in this study.

#### Temporary relocation to natal villages

Some caregivers temporarily relocated to natal villages during the hot season and monsoon, when illness was prevalent, fuel scarce, and flooding hazardous. Rural settings were perceived as offering cleaner air, fewer pests, and stronger family caregiving support. One participant reflected:

Maybe there are some inconveniences during the rainy season here. But in the village, the cool breeze and calm nature make everything feel peaceful. Our children got sick once in Dhaka, and it was hard to manage. That’s when we felt village life was better. (Composite from responses from P04, P16)

At the habitual IBM-WASH level, relocation disrupted the environmental consistency (same kitchen, stove timing, meatsafe location) on which habit formation depends [17, 19, 20], effectively restarting the routine-building process upon return and reducing the likelihood of automated protective behavior.

#### Navigating blame and vulnerability in shared care

Participants not only acknowledged contaminated food, unwashed hands, and open storage as diarrhoeal risks but also cited prenatal malnutrition, seasonal illness, and peer exposure as contributing factors:

Sometimes the child eats with dirty hands or from the floor. Sometimes it’s food left open, or baby food not covered. But also, my child was weak from birth, maybe from what I ate while pregnant. (P12)

Efforts to limit children’s exposure to sick peers were frequently overruled by in-laws. One mother explained:

I try to keep him away from others when they’re sick, but my in-laws say let him play. They feel bad when the child cries. Then I get blamed for being too harsh. (P06)

At the household/interpersonal IBM-WASH level, contested childcare authority diluted maternal motivation to maintain protective routines: when in-laws overruled protective decisions, the social cost of adherence exceeded its perceived benefit.

### Contextual dimension

#### Fuel scarcity and crowded housing

Gas shortages, shared stoves, and crowded living space undermined reheating, water boiling, and utensil cleaning. With five to eight families sharing a stove receiving gas available only intermittently, typically off around 6 p.m. and returning between 7:30 and 9 p.m., reheating leftovers or boiling water during the day was often impossible. One mother explained:

Seven to eight families share one gas stove. There’s no fixed turn, and often no gas at all. We can’t reheat food. We have to feed our children stale food

If there’s no gas, we can’t cook again. The children cry from hunger, but we have to feed them whatever food is left. Sometimes I buy biscuits, but if there’s no money, they eat stale rice. (Composite from responses from P02, P03)

Even if I want to boil water or warm leftover rice, I cannot. The stove has to serve many families. By the time it’s my turn, I’ve already missed the time when the children need to eat or drink. (P02)

High population density exacerbated pest and dust exposure throughout the neighborhood. Although many participants valued the meatsafe for reducing fly and rodent access, ants and small cockroaches still entered through the mesh or when doors were opened, and a few users reported resorting to pesticide inside the cabinet.

#### Seasonal barriers

Summer heat and humidity accelerated spoilage of cooked food and raw ingredients, with food turning sour by midday if cooked the previous night. During the monsoon, flooded courtyards and contaminated floodwater made kitchen access hazardous, prompting batch cooking to reduce stove trips. Batch cooking further extended storage duration and increased spoilage risk. Floodwater also entered homes, contaminating cooking spaces. Caregivers elevated utensils and the meatsafe on bricks or planks, but this offered only partial protection against splashing or pest ingress. One mother noted:

When it rains, rats come in to escape the water outside. They scurry around the floor, trying to get near food. Even if we put the rack on top of bricks, rats sometimes climb up. (P07)

Three dominant contextual pathways emerge. First, energy scarcity eliminated reheating and enforced batch cooking with extended storage, increasing pathogen exposure duration. Second, pervasive environmental contamination pressure from flies, rodents, floodwater, and sewage overwhelmed the partial exclusion that the meatsafe’s mesh walls could offer. Third, seasonal disruption simultaneously accelerated spoilage, lengthened batch-cooking storage periods, and periodically displaced households from Dhaka, interrupting routine use during the highest-risk periods. These pathways interact: energy scarcity drives batch cooking; seasonal heat accelerates spoilage; prolonged storage extends the window of pest and microbial pressure.

## Discussion

The meatsafe was designed as a nudge-based enabling device: a physical object intended to make protective food storage convenient without requiring complex new habits. Application of the IBM-WASH framework across interviews and spot-check records provides plausible explanations for why this logic did not translate into contamination reductions, and what would be required for it to do so. A useful organizing distinction is between fidelity barriers, those that may have prevented execution of the single protective behavioral act the intervention required, and beyond-fidelity barriers, those that may have sustained contamination even with complete execution, as each category maps directly onto different intervention design implications.

### Fidelity barriers

The assumed theory of change required immediate placement of hot food into the meatsafe, with the mesh design alleviating concerns about trapped steam accelerating spoilage while excluding insects and animals, followed by door closure. Spot-check data recorded only whether food was inside the cabinet at the time of visit, and could not capture whether food had been placed there immediately after cooking or left to cool uncovered first. Qualitative interviews revealed a behavioral gap invisible to spot-check monitoring: the distinction between any storage in the meatsafe versus immediate storage.

On the psychosocial side, caregivers continued to be concerned about trapped steam, and left freshly cooked food uncovered outside the meatsafe until cooled, creating the same contamination opportunity that exists in households without meatsafes. This points to a gap in the BCC package: the phrase “immediate covered storage” may have been interpreted as “cover the food once it has cooled,” which is functionally what participants did. They did cover food, and they did use the meatsafe, but only after an uncovered cooling period that was the primary contamination window. IBM-WASH locates such beliefs at the individual level of influence, where they are shaped by habitual experience and resistant to displacement through a single educational contact [8]. On the technological side, rexine liner warping from hot containers also contributed to delay in storage of cooked food.

Additional structural features may have also contributed to cumulative friction. Frame instability on damp, uneven floors required improvised brick stabilization before each use. Latch weakness and frame gaps may have allowed cockroach entry even when doors appeared closed, undermining the mesh exclusion mechanism. Sharp edges made sustained cleaning effortful and, in some cases, caused injury. Cabinet size was further reduced by mixed storage of dry goods, utensils, and personal items, leaving less space for cooked food.

Prior hardware trials used formative research to allow technology modification before deployment [11, 12, 14]. The current intervention drew on formative work in which rural Bangladeshi women expressed demand for meatsafes, and deployed a locally available commercial product without prior controlled testing. The urban informal settlement context of Korail differs substantially from those rural settings, with shared stoves, dense pest pressure, and seasonal flooding posing contamination challenges that the device was not specifically designed or tested to address.

Seasonal relocation to natal villages may have added a contextual fidelity barrier. Caregivers who left Dhaka during the hot season or monsoon may have interrupted whatever storage routines had begun to form, returning to restart that process during the period of highest gastrointestinal illness risk. Habit formation research identifies environmental consistency as a precondition for automaticity, and relocation may have disrupted that consistency at a particularly high-risk time.

Individually each represents a manageable inconvenience; cumulatively, they transformed a nominally simple behavioral act into a multi-step sequence at every cooking event. Research on habit formation suggests that behaviors requiring sustained effort and multiple decision points are less likely to become automatic [17, 19, 20], and the inconveniences expressed by our respondents may well have eroded consistency even among motivated users.

### Beyond-fidelity barriers

The meatsafe was designed to interrupt a specific point in the fecal–oral transmission chain: contact between insects, pests, and cooked food at the point of storage, and potentially hand contact with stored food. It was not designed to address the many other routes through which fecal contamination reaches children. Contaminated water used in cooking or drinking, hands unwashed before food preparation or feeding, floodwater entering cooking spaces, and pest contact with food before or during cooking all remained entirely uncontrolled, and represent potential confounders in attributing contamination outcomes to food storage behavior alone.

The current qualitative inquiry unearthed how these other routes operated in practice. Five to eight families sharing a stove with intermittent gas made batch cooking and extended storage unavoidable, exceeding what passive mesh exclusion could address. Reheating was often impossible. Extreme heat accelerated spoilage, and monsoon flooding contaminated cooking spaces independent of device use.

Social dynamics compounded this. Mothers reported constrained decision-making authority, and in-laws overruled efforts to limit children’s exposure to sick peers. When food hygiene is perceived as only one of many diarrhoeal risks, and when primary caregivers lack household authority, individual-level motivation may not translate into sustained behavior change.

Taken together, these barriers suggest contamination may have persisted even had every fidelity barrier been resolved, pointing to a ceiling effect for hardware-focused interventions with minimal behavior change communication. Echoing the sobering results of recent WASH megatrials, which combined hardware provision with intensive behavior change promotion yet demonstrated little impact on child diarrhoea gains [13, 22, 26], the null result may reflect not simply imperfect implementation but a structural mismatch between the intervention’s scope and the full contamination environment in which it was deployed.

### Revisiting the case for efficacy of meatsafe hardware and nudge-based minimal BCC

This trial evaluated real-world effectiveness before establishing laboratory efficacy for this specific device. The efficacy rationale rested on evidence that freshly cooked food is itself free of contamination [15], and that covered storage reduces contamination risk. In an urban Dhaka slum, rice protected from flies by a net cover had 54-fold lower odds of *E.* *coli* contamination than rice left exposed to flies [21], and uncovered complementary food in rural Bangladesh was twice as likely to show high-level *E. coli* contamination [28]. However, 28% of covered containers remained contaminated even in the net-covered condition [21], suggesting mesh exclusion alone may not eliminate contamination from airborne particles or other sources, and leaving the efficacy assumption for this specific hardware unverified under controlled conditions.

Consistent with this nudge logic, the meatsafe was intended to make protective food storage convenient without requiring complex behavioral instruction. This logic has held where the target behavior is a discrete, low-friction act, as with painted footprints to handwashing stations [7, 10], and food safety-printed children’s plates [2, 6]. Food storage in Korail was embedded in a multi-step behavioral sequence under active pest pressure and structural constraints.

Prior food hygiene trials that achieved contamination reductions, including the Bamako HACCP trials [33, 34], the Dhaka behavior change communication trial [16], and the Malawi Banja la Ukhondo trial [25], all used intensive, repeated promoter contact that directly observed and corrected storage timing. Minimal behavior change communication, as used here, may simply not have been sufficient behavioral dose to produce observable impact, consistent with broader WASH literature finding that hardware alone rarely generates health gains [24].

## Limitations

Findings represent plausible mechanisms rather than prevalence estimates. The purposive sample intentionally oversampled households with documented issues to achieve exhaustive barrier identification, and barriers were in many cases elicited through probing rather than volunteered. Overall device use was high and spot-check compliance was favorable. The connection between the barriers identified here and the trial’s null result is therefore inferential; this substudy was designed to generate hypotheses, not to establish which barriers drove contamination outcomes or in how many households. That question requires quantitative analysis of household-level predictors of *E. coli* detection.

The IBM-WASH framework served as a broad organizing lens for eliciting perception- and practice-related barriers, not a systematic analytical axis; 16 interviews cannot support granular level-domain attribution. All behavioral data rely on self-report and are subject to social desirability bias. The 1-month data collection window may have missed longer-term adaptation or abandonment.

The behavior change communication package advised immediate placement of covered food inside the meatsafe. Whether advising immediate placement without covering would have better resolved the cooling gap remains untested. That message could not be delivered ethically without controlled evidence that uncovered food inside a closed meatsafe stays microbiologically protected, an assumption the trial had not verified. Messaging therefore reinforced covered storage, which may have inadvertently sustained the practice of letting food cool uncovered before placing it inside the meatsafe.

Finally, the qualitative data cannot classify individual households by execution quality or identify which barriers each household faced; heterogeneity in barrier profiles likely contributes to variation in contamination outcomes this substudy cannot fully characterize.

## Conclusion

Using the IBM-WASH framework to identify barriers across levels of influence, this substudy offers plausible explanations for the trial’s null result. Fidelity barriers, including the steam-spoilage belief, liner warping, and frame instability, likely prevented immediate hot-food storage in practice. Beyond-fidelity barriers, including energy scarcity, flooding, and contested childcare authority, may have sustained contamination regardless of device use. Critically, even with full fidelity, efficacy under controlled conditions was never established, and the real-world contamination environment in Korail likely exceeds what passive hardware with minimal behavior change communication can address. Structural improvements to the meatsafe, including liner material that tolerates hot containers and a stronger latch mechanism, and more targeted behavior change communication are warranted, but consistent with recent WASH megatrials, incremental improvements may only go so far without concurrent investment in the structural conditions that shape whether any food safety intervention can work at all.

## Supplementary Information

Additional file 1.

Additional file 2.

## Data Availability

The qualitative transcripts generated during this study and analyzed to support the conclusions of this article are not publicly available due to concerns regarding participant confidentiality in a dense informal settlement context, but de-identified excerpts are included within the article. Additional data may be made available from the corresponding author upon reasonable request.

## Abbreviations

BCC: Behavior change communication
CFU/g: Colony forming units per gram
F-diagram: Fecal–oral transmission diagram
HACCP: Hazard analysis and critical control point
IBM-WASH: Integrated Behavioral Model for Water, Sanitation, and Hygiene
icddr,b: International Centre for Diarrhoeal Disease Research, Bangladesh
PI-1–PI-5: Post-intervention visits 1–5
PVC: Polyvinyl chloride
RA: Research assistant
RCT: Randomized controlled trial
RGHI: Reckitt Global Hygiene Institute
WASH: Water, Sanitation, and Hygiene
