# Supplementary material for: User experiences with food storage in a meatsafe (mesh-covered food storage cabinet) in an informal settlement in Dhaka, Bangladesh
Source: Trop Med Health. 2026 Jun 5;54:188. doi: 10.1186/s41182-026-00983-x (PMC13540818; doi:10.1186/s41182-026-00983-x)
Supplement: Supplementary file 1 [file 41182_2026_983_MOESM1_ESM.docx]

**Supplementary Table S1. Household identifier to participant identifier crosswalk for quoted and cited sources (qualitative sub-study, n = 16 households)**

| # | Quote | Participant |
| --- | --- | --- |
| PSYCHOLOGICAL DIMENSION | "I was telling my husband that if you keep food in a miskin [meatsafe], cockroaches and rats won't get to it. Other people in the slum were also saying, give them a meatsafe. But I cannot buy it on my husband's income — he doesn't earn enough. So how will I buy a meatsafe? Food stays good if you keep it in one — cockroaches don't get to it, rats don't do anything. But I could not buy a meatsafe due to financial reasons." | P01 |
| PSYCHOLOGICAL DIMENSION | "When we used to live in the village, we saw meatsafes in the homes of those who were well-off. But we didn't recognize what it was. When we came back again to Korail slum, we were managing to pay house rent and live. Many people in the slum bought one, but we couldn't. Our income is low. I told my husband, can't you buy one from somewhere? With such little income, how could we buy it?" | P02 |
| PSYCHOLOGICAL DIMENSION | "First of all, we are poor people. We came from the village to Dhaka and took a room in the slum. We have to cook together with ten or twelve people. If you cook curry and put it on the table, the rats eat it all up and ruin it. Why don't you buy a rack? We can't buy it. We don't have that kind of money. That is why we keep food on the floor. People keep their food so nicely and neatly — we can't do that." | P03 |
| PSYCHOLOGICAL DIMENSION | Because they had given a piece of paper. People say MK137 promise so many things with their mouths — taking names from the room, taking names from children's bags — those things don't come true. But when something is # Theme Quote Source given in writing and you read it, the belief is different. Since they said they would give it, they would give it." | P04 |
| PSYCHOLOGICAL DIMENSION | "For many days I had been telling the child's father — buy me a meatsafe, get me one. He wasn't buying it. Then when you came and gave it to us, that made me very happy. This is the truth." | P06 |
| PSYCHOLOGICAL DIMENSION | "I had wanted to buy one. But then you all gave one, so my money was saved. People often don't keep their word — but you came back and gave it." | P08 |
| PSYCHOLOGICAL DIMENSION | "It felt suddenly as if Eid day had come. I've been living here for a long time. At no time do I ever get anything. Suddenly I got one — so for getting it, it felt good." | P09 |
| PSYCHOLOGICAL DIMENSION | "Two years have passed without any means to buy one. There wasn't even a chowki [low bed]. There wasn't even a wardrobe. Cockroaches and mice would eat the food, and we'd eat that spoiled, dirty food afterwards." | P14 |
| PSYCHOLOGICAL DIMENSION | "There was no income at home with which we could buy a meatsafe. If we kept food at home, cockroaches and rats would spoil it. But we couldn't even understand — we'd eat those things. Then it would turn out we had eaten contaminated food. Indigestion and diarrhea would happen, nausea would happen — but we couldn't understand why." | P16 |
| PSYCHOLOGICAL DIMENSION | "After cooking, I cover the pot and keep it in the meatsafe. I cover it with a silver lid, but I leave the lid slightly ajar so the steam can escape. I let out a little steam, then cover it and keep it. I let a little steam out — if food is kept too hot, it spoils. The water from the steam also spoils the food afterwards. The food gets mushy from staying covered too early." | P06 |
| PSYCHOLOGICAL DIMENSION | "If you try to cover hot things, the curry spoils and starts to smell. The rice gets sweaty under the lid. Hot spices easily cause the rice to go mushy." | P08 |
| PSYCHOLOGICAL DIMENSION | "If I put hot food in the meatsafe and cover it with a lid, the food will spoil. I put it down outside, let it cool a little, then take it inside the meatsafe — because steam (literally 'sweat') makes the food spoil. That's why I have to put the food down and let it cool first." | P16 |
| PSYCHOLOGICAL DIMENSION | "The food I keep outside, I keep it to cool down. If mosquitoes and flies fall on that food, I don't understand. I cannot stay there. Because of the child — if mosquitoes and flies fall on it, the food becomes contaminated. Inside the meatsafe I cannot put hot food directly, I have to keep it outside, cover it loosely with a lid, keep a little gap. If mosquitoes, flies, or any insects fall into that food and we eat it, we could have difficulties. I don't know that." | P01 |
| PSYCHOLOGICAL DIMENSION | "I cannot keep hot rice and curry immediately because the plastic catches fire and catches air first — the heat damages the interior. So we ourselves wait seven or eight minutes. When the pot cools down a little, then we put it inside." | P02 |
| PSYCHOLOGICAL DIMENSION | "Cockroaches, garbage — we used to eat them with rice and curry. Road dust got into our food, and cockroaches were found in it. Now everything stays covered, and because of that we eat with no discomfort going down [no illness]." | P03 |
| PSYCHOLOGICAL DIMENSION | "On the cockroach's chest there are things — germs. I don't know what insects eat or don't eat. They eat this too, but they don't throw the food out. There's hot oil on the food and also germs. If you don't follow the rules, you often get sick. If you follow these, there will be no stomach upset." | P04 |
| PSYCHOLOGICAL DIMENSION | "I will not go beyond disease-causing organisms and immunity. The children get sick. That is why we teach them — people will get more illness. We tell them: use the latches and keep them fastened, stay clean, go to one corner of the toilet, wash hands nicely with soap and conduct yourself properly so that immunity remains better." | P02 |
| PSYCHOLOGICAL DIMENSION | "If I myself saw the food had been tipped over, I would throw it away. But when I wasn't there, when I didn't look, I would just feed that food anyway. Then I'd get suspicious — I'd say to myself that something hadbeen tipped over, had something eaten from it or not — I'd get that feeling. I wasn't sure, but I had a guess that this could happen. Say a bit of dirt fell on some food — I pick it up and eat it, but then I myself getsuspicious about it; that thing itself will cause his problem." | P04 |
| PSYCHOLOGICAL DIMENSION | "When my child comes home he gets hungry right away, and then I don't look at the food carefully and just feed him. Whatever the child eats or doesn't eat, the child will get a stomach upset. If I see the child's food is intense [too strong/spoiled], I feed the child the food. What will this food do to the child?" | P04 |
| PSYCHOLOGICAL DIMENSION | "I felt the stomach upset happened due to the heat from the room above. It was very hot up there, and at that time I myself felt bad inside. There was a fever, body heat — the food was getting hot. I thought the stomach upset happened because of this." | P06 |
| PSYCHOLOGICAL DIMENSION | "At night I'd hear sounds [of rats]. From that I could understand that the food had been touched by rats. Then loose stool — the baby used to have it. Later, whatever the rat wanted, there is a mark of it on the food." | P08 |
| PSYCHOLOGICAL DIMENSION | "When food got spoiled, many times I couldn't tell it was spoiled. We would eat it anyway. The day I understood, I understood that the food the baby eats was contaminated. That's why I started keeping the baby's food inside the bucket." | P08 |
| PSYCHOLOGICAL DIMENSION | "When I eat food, I put on the latch. I keep it clean. My neighbors don't do anything like that. They don't keep their food covered. They don't put on the latch. They don't maintain cleanliness. I keep it a bit better. Since I have my child, my family, we're staying away from my child's illnesses. My neighbor's food, even fish, is spoiling — they're suffering from disease germs." | P16 |
| PSYCHOLOGICAL DIMENSION | "Around us there are many neighbour-women. I see them and I tell them: they don't keep food covered, they leave it open, they don't keep food covered inside the meatsafe. I advise them: keep your food covered, keep the latch on. I keep my food covered. The neighborhood women around don't keep it like this. We don't have that much financial capacity to go to the doctor again and again. Loose stool, vomiting — these things won't happen if you follow what I do." | P11 |
| PSYCHOLOGICAL DIMENSION | "We push and teach them: conduct yourselves nicely,keep it clean — use soap in the toilet, wash the childwith soap while bathing, and yourself too — before and after eating, if you touch any dirty thing, use soap. We teach these things to those who don't understand." | P02 |
| PSYCHOLOGICAL DIMENSION | "I have seen at the houses of people around me — Gholam Mohammad's [neighbor's] meatsafe is sturdy, big, good from every side. He can keep everything, things stay with a boundary, water doesn't fall in. Like inside mine — I cannot keep hot food at all. The food's water falls at the bottom. The latch is small, I can't fasten it. There is nothing steel-type inside. The gaps are big, dust enters." | P01 |
| PSYCHOLOGICAL DIMENSION | "Those who bought their own meatsafes, with money — the ones with good latches — they kept them. They looked at whether the appearance was sturdy or whether it had gaps before buying. The cholera hospital gave us whatever was available. Gaps and holes — that's the thing. The latch is just a normal plastic one. It doesn't keep food properly. But those who bought their own — theirs are indeed sturdy. They don't keep things properly though, because they didn't receive any advice. We are incomparable to before [we have improved because of the guidance]." | P02 |
| PSYCHOLOGICAL DIMENSION | "Many people, when given a lot of papers, just take photocopies, like the child's vaccination papers. They take them but they don't read. We can't talk to them. But we can talk with you all. When you talk with a person, they believe what you say. They gave papers — I didn't believe those either. But you come home and talk, you give assurance, you give paper. That is belief." | P04 |
| PSYCHOLOGICAL DIMENSION | "Writing it on paper has been good — because not everyone understands otherwise. They could understand by looking at pictures, but not everyone can read." | P06 |
| PSYCHOLOGICAL DIMENSION | "Some don't know how to read and write. Those who can't read will not understand this writing. If told verbally, I'll understand." | P10 |
| PSYCHOLOGICAL DIMENSION | "If you had given a few pictures, I could have learned many things and understood. I don't know how to read and write." | P13 |
| PSYCHOLOGICAL DIMENSION | "My mother-in-law would scold me — 'How are you feeding the child? What are you feeding?' I would say, I cooked and kept it properly. But what's happening, I don't know about that. The child is not always at home — he stays at my mother-in-law's room. When he comes home he gets hungry right away, and then I don't look at the food carefully and just feed him." | P06 |
| PSYCHOLOGICAL DIMENSION | "I try to keep him away from others when they're sick, but my in-laws say let him play. They feel bad when the child cries. Then I get blamed for being too harsh." | P06 |
| PSYCHOLOGICAL DIMENSION | "Sometimes the child eats with dirty hands or from the floor. Sometimes it's food left open, or baby food not covered. But also, my child was weak from birth, maybe from what I ate while pregnant." | P12 |
| PSYCHOLOGICAL DIMENSION | "We have a chest problem — sometimes the pain comes back. When the pain comes I feel like I'm leaving this world. Such pain rises. When the gastric pain got worse I used to take medicine. There is weakness in the body." | P12 |
| PSYCHOLOGICAL DIMENSION | "Father is cleaner than me. At the office, all of that — handwash, handwash, gets it everywhere. Habit of putting hands under the basin. If he comes home, he explains it." | P04 |
| PSYCHOLOGICAL DIMENSION | "My child bothers me a little. There's no one else there for her. If I go to the washroom, she cries. She goes to someone else and cries until she sees me. Then I have to rush to go." | P04 |
| PSYCHOLOGICAL DIMENSION | "The children make excuses. Always. Even when I tell them to wash their hands, they make excuses." | P13 |
| PSYCHOLOGICAL DIMENSION | "I myself know, and I can let others know too — that cleanliness and hygiene are good for everyone's survival." | P13 |
| PSYCHOLOGICAL DIMENSION | "Some keep it without cleaning it. I do it differently — I clean it. But I study, I try to learn." | P12 |
| PSYCHOLOGICAL DIMENSION | "Come and see how I am keeping things in my home.If you keep it nicely, cockroaches and mice can't get in.with home demonstration Then keep everything nicely. You won't fall sick anymore. The child won't have diarrhea." | P07 |
| PSYCHOLOGICAL DIMENSION | "It has become much better. We have benefited. But we are slum people, and what I got, I cannot say is fully what I needed." | P01 |
| PSYCHOLOGICAL DIMENSION | "Before, I used to cook once and eat repeatedly. If there was a gas problem, I'd cook once and keep it all. This system of cooking once and eating twice didn't exist before getting the meatsafe. Now I can cook, leave it for one meal, then the next time we eat. Before, I didn't trust the food to last." | P06 |
| PSYCHOLOGICAL DIMENSION | "By keeping the child's food inside the meatsafe, I remember that I have kept the food there. Before, I used to scatter it here and there. Now I don't scatter it anymore." | P04 |
| PSYCHOLOGICAL DIMENSION | "I couldn't quite like it at first — beautiful, yes, but no flowers, no decorative design. Later, though, I came to appreciate it." | P13 |
| TECHNOLOGICAL DIMENSION | "The latch needs to be fastened, but there are gaps. My husband put cello tape inside it to try to close them. All around the inside there are gaps. It has been taped. Even if I fasten the latch, there are still gaps." | P01 |
| TECHNOLOGICAL DIMENSION | "The latch is fragile. It broke automatically. There is no arrangement for a lock. When children push it, after cooking and bringing food in, you can feel there is a gap. It has to be pushed in again. If the latch could be fastened all at once, if there were a lock arrangement, we could keep all things properly closed." | P02 |
| TECHNOLOGICAL DIMENSION | "You gave us one, but the latch cannot be fastened. Even then, hot food cannot be kept inside. The latch-cutting problem — it cannot be fastened. It is very difficult to fasten it. If I could get a good rack, all things could be kept inside." | P03 |
| TECHNOLOGICAL DIMENSION | "The joint has opened up a little there. I had asked the repairman to come, but the repairman didn't come." | P05 |
| TECHNOLOGICAL DIMENSION | "It is also difficult to latch the latch. It is stiff and heavy, and there is a gap. Making it very tight makes it very problematic. The cloth [fabric panel] is very tight but there is still a gap. I myself fix it and put it on. The meatsafe latches get bent over time." | P07 |
| TECHNOLOGICAL DIMENSION | "My child's finger gets in and then he opens it. He is a bit too naughty. Then he throws those things out and takes them outside. What I'm saying is that here I could put on a lock, because the baby is small and does what he does." | P08 |
| TECHNOLOGICAL DIMENSION | "My child keeps pulling it open and opening it repeatedly, so I can't lock it. He pulls open the meatsafe by yanking at it. If you had an arrangement for a lock, it would be good — so I could lock it up and go here and there [leave the house]. I can't lock it." | P11 |
| TECHNOLOGICAL DIMENSION | "There are quite a lot of gaps. Cockroaches and geckos get into the mesh panels. The door opens up on its own at the top. Sometimes the latch snaps open. Hot food I can't bring and put directly inside — the rexine will melt. From the very first day I saw it had fallen. Then it fell onto the bed." | P12 |
| TECHNOLOGICAL DIMENSION | "There's a small problem inside. I can't latch the bolt. If you had applied a little pressure to fix it when you brought it, it would have been a little better. The bolt has come loose — it can't be fastened. Sometimes cockroaches and mice get inside it too. If you had checked it and given it, it would have been a bit better for us." | P14 |
| TECHNOLOGICAL DIMENSION | "I can't put the latch on properly. If the latch were a little bigger, then it could be put on properly." | P16 |
| TECHNOLOGICAL DIMENSION | "There are quite a lot of gaps. Small cardamom seeds fall through — that's how big the holes are. Cockroaches. Geckos. They get in through the mesh panels." | P12 |
| TECHNOLOGICAL DIMENSION | "Even when closed, a 2–3 finger-wide gap remained between the door and the frame, through which cockroaches and small pests could enter." | P12 |
| TECHNOLOGICAL DIMENSION | "A small cockroach entered my meatsafe and I saw it and took it out. There is a gap through which a cockroach could enter. The top part — like a little room they put there — also has a gap. The whole place is full of gaps. Even if I fasten the latch, there are still gaps." | P01 |
| TECHNOLOGICAL DIMENSION | "The problem is that the meatsafe has holes — sand and dust get in through the steel mesh. Cockroaches, rats, and small pests get in through these. All the cockroaches of the slum — small ones, and geckos — get in because the openings are large." | P11 |
| TECHNOLOGICAL DIMENSION | "The mesh should be a little finer, that would be better. There are ants in this. And small baby cockroaches — there would be no chance for them to get in if the mesh were finer." | P04 |
| TECHNOLOGICAL DIMENSION | "I cannot bring hot cooked food and put it inside directly. I cannot keep it because the plastic will melt. The inside is too soft. After the food cools down, then I keep it inside. If they had given something made of steel, something solid, then I could have kept hot food in it." | P01 |
| TECHNOLOGICAL DIMENSION | "I can't keep hot rice and curry immediately. The place is not good — it catches fire, catches air first [the hot food damages the plastic interior]. After seven or eight minutes, when it cools down a little, then I have to put it inside. If it were made of steel, it would be convenient for us." | P02 |
| TECHNOLOGICAL DIMENSION | "If hot food is kept inside, the rexine will melt. Without a steel base, the rexine is melting and I can't keep food. Hot food in the rexine — the smell of food gets absorbed into it." | P11 |
| TECHNOLOGICAL DIMENSION | "If they had given a steel base underneath the plastic, it would have been of use. Because of the plastic, when hot food is placed, the plastic melts directly. If they had given that lid, I could have used it. After cooling, I put the food inside.Until then, I have to keep it outside." | P07 |
| TECHNOLOGICAL DIMENSION | "If your meatsafe were steel, then I could have kept hot food well. Because when steam and heat hit the plastic, it can melt. That's why we couldn't keep hot food. Because of the plastic, I can't keep hot food — it would spoil." | P16 |
| TECHNOLOGICAL DIMENSION | "There are small protrusions inside — when I go to clean, my hand gets cut. Cockroaches and rats are getting inside the meatsafe. Our food is maybe spoiling a little, we can't even understand. Even so, we try to maintain cleanliness. I clean once a month. Even then, there's so much dust in the slum, so much spoils. Cleanliness doesn't stay." | P16 |
| TECHNOLOGICAL DIMENSION | "If you wipe it, your hand gets cut. There are many problems — dirt stays inside the corner, we can't take it out. We can't keep food inside properly." | P03 |
| TECHNOLOGICAL DIMENSION | "When I go to wipe it once a week, my hand gets cut, my hand has problems. Or when I want to put the bolt back on, there's a lot of trouble." | P14 |
| TECHNOLOGICAL DIMENSION | "We clean once a week, with water. Still I can't keep this clean. I clean it and then from the gaps dust will enter. Even when you clean, dirt still remains inside. It doesn't stay good. There is nothing steel-type inside. That is why I can't keep things [clean]." | P01 |
| TECHNOLOGICAL DIMENSION | "Every two to three days I take everything down and wipe it with rexine cloth. Every fifteen days I do a more thorough clean." | P04 |
| TECHNOLOGICAL DIMENSION | "On Friday, I take two drops of Savlon and with soap, I wipe the plastic parts nicely and clean. But the plastic becomes soft like that. Soft. It becomes that condition over time." | P07 |
| TECHNOLOGICAL DIMENSION | "If I put something heavy at the bottom, it tilts over. I cannot keep food. I still have to keep things outside. Large pots cannot be kept inside. These are completely lightweight. There are gaps inside." | P01 |
| TECHNOLOGICAL DIMENSION | "We put bricks underneath, otherwise it shakes. The floor isn't level, and when you open the door, it can tip forward." | P12 |
| TECHNOLOGICAL DIMENSION | "During rain, we have almost no gas for cooking. Water rises, and water enters the gas line, so it is difficult for us to cook. Then we cook and have to keep it [the meatsafe] elevated. When it rains, you have to collect the food and keep it elevated. You have to keep it inside the meatsafe. But still there are mosquitoes and flies — through the gap, they can enter." | P01 |
| TECHNOLOGICAL DIMENSION | "They said to wash with clean water and keep [the utensils] inside the meatsafe. I cannot do this. If I keep that water in the meatsafe, then the water falls into my other food. If I keep something on top, water will fall on the food below. If I cook and keep food in one compartment, dry food in another — water will fall on it." | P01 |
| TECHNOLOGICAL DIMENSION | "Inside, I keep rice and curry, dry food, the child's khichuri, noodles, and when I warm the child's milk, I keep that too. I also keep potatoes on one side, like a fridge." | P02 |
| TECHNOLOGICAL DIMENSION | "I am fine with it in every way. No insects can get in. I keep all our food, the child's food, our cooked food, dry food. There are all sorts of things for the child's food, medicine, everything." | P04 |
| TECHNOLOGICAL DIMENSION | "Some households repurposed the meatsafe for non-food items such as clothes, medicines, or baby supplies, alongside food and utensils." | P04 |
| TECHNOLOGICAL DIMENSION | "I store biscuits, raw vegetables, powdered milk, spices, oil bottles, and utensils alongside cooked food." | P06 |
| TECHNOLOGICAL DIMENSION | "Before receiving the meatsafe, participants stored cooked food on the floor, on tables and stools, under beds, in jute sacks, or on improvised wooden racks, often inside metal containers or buckets." | P03 |
| TECHNOLOGICAL DIMENSION | "If the meatsafe were a little bigger, with the shelves having two or three more compartments, it would have been better. I cannot keep vegetables and those things inside. I cannot keep all the dry things — I have to keep them outside." | P01 |
| TECHNOLOGICAL DIMENSION | "If a brush were given so I could clean it, I could wash it with a brush. Right now I clean it with a broom — but the dirt from the broom gets pushed inside through the holes. We need a brush." | P11 |
| TECHNOLOGICAL DIMENSION | "The paper liner tears when it gets wet. Then cockroaches come. So I use cloth instead. I cut up my orna [traditional woman's scarf] and put it on the shelves. After that, rexine [PVC-coated fabric] became the most durable option for shelf liners." | P04 |
| TECHNOLOGICAL DIMENSION | "Food was sealed at intermediate temperatures — characterized in field-visit notes as 'not burning hot but still warm.' The interior environment was warm enough to permit microbial proliferation inside closed containers." | P02 |
| TECHNOLOGICAL DIMENSION | "Several households reported applying insecticide inside the cabinet to address recurrent pest intrusion through structural gaps. This adaptation introduces a chemical contamination risk to stored food." | P01 |
| CONTEXTUAL DIMENSION | "We live in a slum house. Our income is low. My husband drives a rickshaw. It is difficult to run the household with that money. Paying rent is difficult for us." | P01 |
| CONTEXTUAL DIMENSION | "And five people are living on one person's income. There is a lot of going about for us — movement, food, clothing, dress — all problems." | P02 |
| CONTEXTUAL DIMENSION | "My husband earns alone — about 10,000 taka per month. With that he sees the doctor like a king. The child's food, our own food, the child's food from the shop — that causes many problems. It's difficult." | P14 |
| CONTEXTUAL DIMENSION | "Health guidelines, soap — I can't afford to buy soap. I can't buy things for my daughter. I can't buy things for my mother." | P12 |
| CONTEXTUAL DIMENSION | "Within one gas stove, seven or eight families cook.Once you put food, it has to last the whole day. There is no time to reheat. You can't get a turn — there is no gas. A lot of problems with gas. So if this meatsafe were a little bigger, that would be better, because we have to store everything from one cooking session." | P02 |
| CONTEXTUAL DIMENSION | "We can't get a turn at the stove. Many times we can't reheat. We feed the children that stale food. If the child goes without eating, the child cries. That is why we feed more stale food to the child. If you eat rotten stale food, diarrhea or cholera comes back. The child eats it because of hunger — that is why." | P02 |
| CONTEXTUAL DIMENSION | "Now Tahera cooks on the stove and screams and shouts. Cannot tolerate. So many times they don't let us light it, because then the child's mother can't breathe. Fights and arguments happen. One person's smoke — another person has to tolerate. That is why they don't let us light it." | P02 |
| CONTEXTUAL DIMENSION | "If we buy something with money and bring it from the shop, or we bring muri [puffed rice] and eat it. Nothing else can be done." | P02 |
| CONTEXTUAL DIMENSION | "There is no gas. We have to take turns — five of us anyway. The child cries. Problems are always there for us." | P03 |
| CONTEXTUAL DIMENSION | "Eleven families share the stove. If you cook for all of you, it takes one hour, half an hour just to wait for gas. Around six o'clock the gas goes. Again around seven-thirty or nine o'clock it comes back. Boiling water is impossible. The children bring water from downstairs. I can't boil water." | P12 |
| CONTEXTUAL DIMENSION | "Because of the gas problem, once I cook properly, I can't heat it again and eat properly a second time. With the gas problem, cooking a second time doesn't happen. In Korail slum, once you've cooked, if you go a second time to the stove, there's a quarrel over it every time. Once cooking — somehow, greens, dal, rice — those have to be stored properly. If not stored, we can't eat it again." | P11 |
| CONTEXTUAL DIMENSION | "Gas is not available. We make effort in our cooking. We cook on the lucky stove [a portable stove]. This difficulty is what happens to us, from morning till night." | P07 |
| CONTEXTUAL DIMENSION | "Even if I want to boil water or warm leftover rice, I cannot. The stove has to serve many families. By the time it's my turn, I've already missed the time when the children need to eat or drink." | P02 |
| CONTEXTUAL DIMENSION | "We live in a slum. One tap. Thirty families. Then two bathrooms. With this many families, how can we fully follow the rules? Water problem, gas problem — there are many problems. They stay unclean. Cleanliness doesn't stay. We can't store water. There's no gas." | P16 |
| CONTEXTUAL DIMENSION | "Water — I bring it from downstairs and cook with it. There's a problem lifting water upstairs in pots and pitchers. We live on the second floor and the water tap is below." | P12 |
| CONTEXTUAL DIMENSION | "When there's no gas, we get water from somewhere else, from another place. The water smells [is not clean]." | P11 |
| CONTEXTUAL DIMENSION | "Inside one toilet, forty, fifty — meaning a hundred people use one toilet. When I go to that bathroom, even a healthy child's stool is not good — they get sick. Toilet is not clean. Many families use the same toilet. That is why we have a lot of problems, more illness.""sick. Toilet is not clean. Many families use the same toilet. That is why we have a lot of problems, more illness.""" | P02 |
| CONTEXTUAL DIMENSION | "Have to go to the bathroom in turns with four or five people. There are fights. Then things stay dirty." | P03 |
| CONTEXTUAL DIMENSION | "Eleven families share one toilet. The bathroom isn't kept clean either. Once a month — washing and wiping — that's the cleaning frequency for eleven families." | P12 |
| CONTEXTUAL DIMENSION | "One bathroom inside, everyone uses it. Because of the tap, you have to press the hand pump. There are many queues. One toilet, for thirty people. With so many people in the slum, I can't follow the rules." | P14 |
| CONTEXTUAL DIMENSION | "In the slum, because of the water problem, we cannot even pray properly [referring to ritual ablution before Islamic prayer]. Even if we want to stay clean, we are not able to." | P02 |
| CONTEXTUAL DIMENSION | "One room — inside this room — cooking inside the kitchen, eating, the child. If everything is in the same room, it's hard to keep clean. Eating inside the room, cooking a little inside, the child and everything — all in a little space. Even if we want to stay clean, we can't manage." | P02 |
| CONTEXTUAL DIMENSION | "I live in one room inside the slum. My room space is small. If the meatsafe were a little bigger, I could keep food in it — but my room is small. Keeping it in a small room is a problem." | P11 |
| CONTEXTUAL DIMENSION | "Rats, mosquitoes, cockroaches, bedbugs, dirt and garbage — these are everywhere. In the slum there is more dirt and garbage. The dust comes and spoils the food. Mosquitoes and flies are too many. No matter how much you cover things, inside the slum there is too much dirt and garbage." | P02 |
| CONTEXTUAL DIMENSION | "The brick kilns [operating near the slum] — dust enters. I keep food outside [to cool], and whether mosquitoes and flies fall on that food or not, I don't know." | P01 |
| CONTEXTUAL DIMENSION | "Loose motion, diarrhea, worms keep on coming.We live inside an unclean house where we can't boil water and drink." | P03 |
| CONTEXTUAL DIMENSION | "In the rainy season, lots of water. Knee-deep water. Can't buy food and eat. Can't go to the market. Can't cook. We don't want to stay with only one meal. In the rain, there's no roof above. Rainwater falls — so we have to cook when the rain stops." | P16 |
| CONTEXTUAL DIMENSION | "When it rains, we work very hard — the water enters the house and we have to hold the child in our lap. In the kitchen we have problems. The road is high, the house is low — water stays. There is garbage. Five people live together. Mosquitoes and flies are always there." | P03 |
| CONTEXTUAL DIMENSION | "In the water comes filth — dirty garbage. The children — inside the house, it's not possible to stay with feet in it. In the water-logged place one cannot stay. These insects come and enter the house — problem keeping the children. Cockroaches and mice fall onto the dry food above, the dishes." | P07 |
| CONTEXTUAL DIMENSION | "When it rains, rats come in to escape the water outside. They scurry around the floor, trying to get near food. Even if we put the rack on top of bricks, rats sometimes climb up." | P07 |
| CONTEXTUAL DIMENSION | "We stay outside because inside the house it is very hot. We can't tolerate it. We sit on the road. Many times we sleep with the door open. Rice and curry gets spoiled and is fed to the children." | P03 |
| CONTEXTUAL DIMENSION | "In this heat, I stay outside — I walk around outside. We live in the slum in a tin-shed house. It's very hot. Rather than staying in the sun, we walk around outside. On hot days, more people get sick — cold, cough, diarrhea — because we catch more disease germs from a lot of dust." | P16 |
| CONTEXTUAL DIMENSION | "In summer, food cooked the previous evening turned sour by midday. Caregivers preserved cooked rice in water — known as panta bhat — as a low-cost preservation method." | P02 |
| CONTEXTUAL DIMENSION | "We eat panta bhat [water-soaked rice] in summer. With our low income we can't eat much. We keep cooked rice with water so it does not spoil too quickly." | P02 |
| CONTEXTUAL DIMENSION | "When the heat is too much, we go to the village. The village isn't this hot — there are trees. The village stays cool. The way we live in the slum, there are tin houses, no trees. There's lots of sun in Dhaka. Mosquitoes, flies, germs are very many in Dhaka. In the village, there aren't as many. So many of us go to our home village so the children can stay cooler.— natal village Five months ago, when diarrhea happened, I took the children to the village." | P16 |
| CONTEXTUAL DIMENSION | "When someone gets very sick, I think — mother is there, sister is there — I'll go to look after them and they'll look after me. If a child is sick, I go home to the father's house. The children are crying. You're sick yourself. In Dhaka, you have to manage on your own. But there, mother and sisters are there. They will help." | P04 |
| CONTEXTUAL DIMENSION | "There is no gas. Then my office responsibility — I leave the child with my mother. I used to take leave from the garments factory and run around with the child. Now I don't need leave as often." | P07 |
| CONTEXTUAL DIMENSION | "For working mothers, food prepared and stored under the meatsafe protocol was often fed by other caregivers — grandmothers, mothers-in-law — in different rooms and under different hygiene conditions, creating a post-storage feeding pathway the device cannot reach." | P07 |
| CONTEXTUAL DIMENSION | "After eating that food, my child had loose stool four times. Later I took her to the Cholera Hospital. She was vomiting. I was admitted with the child for treatment." | P11 |
| CONTEXTUAL DIMENSION | "Hands, feet, eyes, face — turned blank, became strange somehow. I poured water on the child's head and took the child to the doctor. They gave medicine. Cough. Fever. For three days the child was a bit far [from well]." | P12 |
| CONTEXTUAL DIMENSION | "We suffered from diarrhea and cholera for many days. Loose stool. Cholera. I was a hospital patient with the child. Diarrhea." | P16 |
| CONTEXTUAL DIMENSION | "He had eaten chatpoti [a spicy street snack] with me one day, and his father gave him bhelpuri. The day after, he had loose stool. He had also eaten noodles. The mango juice I had bought — a 20 taka packet — he hadn't had before. Because he ate it for the first time, the problem happened." | P08 |
| CONTEXTUAL DIMENSION | "A few users reported resorting to pesticide inside the cabinet to address recurrent pest intrusion through structural gaps." | P01 |
| CONTEXTUAL DIMENSION | "We try to stay clean. All the things the sisters told us, we cannot fully follow. We live inside the slum. There will be filth with us. We cannot follow. We live in a dirty place. Water problem, gas problem — because of these problems, even if we want to wash one thing, we cannot. We cannot cook like rich people." | P02 |
| CONTEXTUAL DIMENSION | "I can't follow the hygiene rules fully. We live in the slum. There isn't enough gas to boil and drink water, and then to cook the food. Why I can't do it — we live in the slum. One tap, thirty families, two bathrooms. How can I follow the rules?" | P16 |
| CONTEXTUAL DIMENSION | "Everyone is in this condition. With how much hardship we keep going. He means — no convenience. The thing won't last many days. Many people think — even they have become a group, the meatsafe has become soft. It cannot all be washed.Nothing can be done." | P07 |
| CONTEXTUAL DIMENSION | "We collect firewood from inside the slum when there's no gas. Cooking is difficult. There is a water problem. Because of various troubles, staying in a flat. The children eat well. I have learned the rules." | P11 |
| CONTEXTUAL DIMENSION | "The brick kilns operating near the slum produce dust that enters all open food. Cockroaches and dust enter through the meatsafe gaps." | P01 |
| CONTEXTUAL DIMENSION | "Inside the house — the road is high, the house is low. Water stays. Five people live together. Mosquitoes and flies are always there. We try to stay clean but we can't, because we live inside the slum." | P03 |
| CONTEXTUAL DIMENSION | "Before, I had to take leave from the garments factory frequently — for the child's illnesses. Now I don't need leave as often. The child stays well. The | P07 |
