# Supplementary material for: User experiences with food storage in a meatsafe (mesh-covered food storage cabinet) in an informal settlement in Dhaka, Bangladesh
Source: Trop Med Health. 2026 Jun 5;54:188. doi: 10.1186/s41182-026-00983-x (PMC13540818; doi:10.1186/s41182-026-00983-x)
Supplement: Supplementary file 2 [file 41182_2026_983_MOESM2_ESM.docx]

**COREQ 32-Item Checklist — Supplementary File 2**

*Manuscript: User experiences with Food Storage in a Meatsafe (Mesh-Covered Food Storage Cabinet) in an Informal Settlement in Dhaka, Bangladesh*

Rahman MJ, Nurunnahar S, Rahman M, Kwong LH, Winch PJ | Line numbers refer to RahmanMJ_MeatsafesRCT_BD_4May2026_CleanVersion.docx as displayed in Word. N/A = item not applicable or not reported in the manuscript.

| **Domain / Topic** | **Item No.** | **Guide Question / Description** | **Line No. in Manuscript** |
| --- | --- | --- | --- |
| **DOMAIN 1: Research Team and Reflexivity** | | | |
| ***Personal Characteristics*** | | | |
| Interviewer / Facilitator | 1 | Two Bengali-speaking RAs conducted in-home, semi-structured interviews. MJR supervised data collection but was not present during main interviews. | Lines 188–191 |
| Credentials | 2 | RAs held bachelor's degrees in public health or social science. MJR holds a PhD in International Health (Johns Hopkins Bloomberg School of Public Health). | Lines 191–192; Authors' information (~line 594+) |
| Occupation | 3 | MJR: public health researcher (JHBSPH doctoral graduate). RAs: community health field researchers employed by icddr,b. | Lines 189–192; Authors' contributions (lines 589–590) |
| Gender | 4 | Gender of RAs not reported in the manuscript. All 16 primary caregivers were adult women (line 160). | *N/A — not reported in manuscript* |
| Experience and Training | 5 | RAs had prior qualitative research experience in community health settings and completed a three-day training covering the interview guide, probing techniques, audio recording, field note conventions, and ethical protocols including informed consent and confidentiality. | Lines 192–195 |
| ***Relationship with Participants*** | | | |
| Relationship Established | 6 | All participants were enrolled in the parent RCT's intervention arm before qualitative recruitment. Field staff had conducted fortnightly visits to participant households throughout the trial prior to qualitative data collection. | Lines 146–147, 157–158 |
| Participant Knowledge of the Interviewer | 7 | Participants knew RAs were affiliated with the icddr,b research team conducting the parent trial. MJR was not present during main interviews. | Lines 190–191 |
| Interviewer Characteristics | 8 | MJR is a Bangladeshi public health researcher with prior icddr,b experience in Dhaka informal settlements. Advantages include language and cultural familiarity and rapport with field staff; risk of over-familiarity managed through analytical memos, regular team debriefs (SN, PJW, LHK), and triangulation against 684 spot-check observations and RA field notes. | Lines 231–237 (Reflexivity) |
| **DOMAIN 2: Study Design** | | | |
| ***Theoretical Framework*** | | | |
| Methodological Orientation and Theory | 9 | Theory-driven qualitative inquiry organized by the IBM-WASH framework (Dreibelbis, Winch et al. 2013), with thematic analysis across three domains (technological, psychosocial, contextual). Prospectively planned barrier-probing mechanism-of-impact inquiry nested within a parent RCT. | Lines 103–118, 212–223 |
| ***Participant Selection*** | | | |
| Sampling | 10 | Purposive sampling based on fortnightly spot-check observational data and laboratory E. coli results from the parent RCT, selecting for variation in meatsafe condition, maintenance profiles, and food contamination levels. | Lines 162–164 |
| Method of Approach | 11 | Research assistants approached candidate households in person and by telephone. | Lines 171–172 |
| Sample Size | 12 | 16 in-depth interviews; one per household; one primary caregiver per interview. No household contributed more than one interview. | Lines 173–175 |
| Non-Participation | 13 | Of 133 households in the issue-identified pool, 13 consented to participate; refusal count and reasons not reported in detail. Three additional no-issue households recruited for a total of 16. Three households had one or two missed post-intervention visits due to temporary absence (not interview refusals). | Lines 172–175, 178 |
| ***Setting*** | | | |
| Setting of Data Collection | 14 | All interviews conducted in the participant's primary domestic space, typically a single- or two-room corrugated iron structure in Korail, Dhaka, containing the meatsafe. Data collected 15 November to 15 December 2024. | Lines 149–150, 196–197 |
| Presence of Non-Participants | 15 | Not explicitly reported. Interviews took place in participants' primary domestic space; presence of other household members during interviews is not described. | *N/A — not reported in manuscript* |
| Description of Sample | 16 | Full sociodemographic profiles for all 16 participants in Table 1: age (range 18–40 years), relationship to index child (15 mothers, 1 aunt), household size, education, PI visits attended, meatsafe condition (door problems, screen holes, dirt observed), and E. coli contamination category (None / Low / High). | Table 1 (after line 248; table caption at ~line 248 in manuscript) |
| ***Data Collection*** | | | |
| Interview Guide | 17 | Semi-structured guide developed iteratively by MJR using IBM-WASH domain definitions and formative knowledge of Korail; reviewed by all co-authors. Pilot tested in 2 households in an adjacent non-intervention area of Korail with similar socioeconomic and housing characteristics, not enrolled in the parent RCT. Pilot interviews conducted by MJR together with one RA. Three probes reworded before main data collection. Pilot data not included in findings. Final guide in Supplementary File 1. | Lines 202–209 |
| Repeat Interviews | 18 | Not conducted. One interview per household; no follow-up interviews. | *N/A — not reported in manuscript* |
| Audio/Visual Recording | 19 | All interviews audio-recorded with participants' informed consent. Recordings transcribed verbatim, translated into English, and reviewed by bilingual researchers for fidelity. | Lines 202, 210–211 |
| Field Notes | 20 | RAs recorded structured observational field notes on meatsafe condition, placement, shelf contents, and visible adaptations (liners, stabilizing bricks, taped seams) immediately after each interview. Notes served as a triangulation source alongside transcripts and spot-check data. | Lines 198–201 |
| Duration | 21 | Interviews lasted 30 to 60 minutes. | Lines 188–189 |
| Data Saturation | 22 | Thematic saturation reached by interview 16. New themes emerged through interview 12; interviews 13 through 16 yielded only minor elaborations on established themes. | Lines 184–186 |
| Transcripts Returned | 23 | Transcripts were not returned to participants for comment or correction. De-identified excerpts are included in the manuscript. | *N/A — not reported in manuscript* |
| **DOMAIN 3: Analysis and Findings** | | | |
| ***Data Analysis*** | | | |
| Number of Data Coders | 24 | Two coders: MJR and SN, independently coding each transcript with collaborative resolution of ambiguities as they arose. | Lines 212–216 |
| Description of the Coding Tree | 25 | First-pass code of 'barrier' applied to relevant segments; coded segments sorted into three IBM-WASH domains (technological, psychosocial, contextual) and grouped into subthemes through iterative comparison. Full quote-to-code-to-theme mapping provided in Supplementary Table 1. | Lines 212–219 |
| Derivation of Themes | 26 | IBM-WASH domains defined a priori as the organizing structure; subthemes derived inductively from coded segments through iterative comparison within each domain. Level attributions (individual, household/interpersonal, habitual) applied only where the data directly support them. | Lines 103–111, 216–223 |
| Software | 27 | NVivo version 14 (Lumivero, Denver, CO, USA) for qualitative data management and coding. Stata version 18 (StataCorp, College Station, TX, USA) for descriptive analysis of spot-check data. | Lines 214–215, 224–225 |
| Participant Checking | 28 | Not conducted. Findings were not returned to participants for feedback or member-checking. | *N/A — not reported in manuscript* |
| ***Reporting*** | | | |
| Quotations Presented | 29 | Participant quotations presented throughout Results to illustrate all themes, each identified by participant ID (e.g., P12) or labeled 'Composite from responses from [P##, P##].' Examples: P12 (line 275), P01 (lines 287–289), composite P01/P02/P11 (lines 290–293), composite P01/P06 (lines 300–302), composite P03/P14 (lines 308–310), P04 (lines 316–317), composite P01/P02 (lines 321–324), composite P06/P08/P16 (lines 329–333), composite P04/P16 (lines 341–344), P12 (lines 353–355), P06 (lines 358–360), composite P02/P03 (lines 372–374), P02 (lines 375–377), P07 (lines 390–392). | Lines 275–392 (throughout Results) |
| Data and Findings Consistent | 30 | Findings triangulated across three data sources: in-depth interview transcripts, structured RA field notes, and spot-check observational data (684 pooled observations from 145 households). Triangulation described explicitly in Data Collection and Analysis (lines 198–201, 224–226) and Reflexivity (lines 236–237). | Lines 198–201, 224–226, 236–237 |
| Clarity of Major Themes | 31 | Three major IBM-WASH domains organized as clearly labeled section headers: Technological Dimension (line 269), Psychosocial Dimension (line 325), Contextual Dimension (line 364), each with named subthemes as sub-headers. Fidelity barriers (line 411) and beyond-fidelity barriers (~line 460) further organize the Discussion. | Lines 269, 325, 364 (Results headings); lines 401–411 (Discussion) |
| Clarity of Minor Themes | 32 | Minor themes addressed within domain subsections: child tampering with latch (lines 294–295), pesticide use inside cabinet (lines 379–381), monsoon-driven seasonal displacement (lines 337–348). Full quote-to-code-to-theme mapping including minor themes in Supplementary Table 1. | Lines 294–295, 337–348, 379–381; Supplementary Table 1 |
